# A Metagenomics Investigation of Carbohydrate-Active Enzymes along the Gastrointestinal Tract of Saudi Sheep

**DOI:** 10.3389/fmicb.2017.00666

**Published:** 2017-04-20

**Authors:** Saad Al-Masaudi, Abdessamad El Kaoutari, Elodie Drula, Hussein Al-Mehdar, Elrashdy M. Redwan, Vincent Lombard, Bernard Henrissat

**Affiliations:** ^1^Department of Biological Sciences, King Abdulaziz UniversityJeddah, Saudi Arabia; ^2^Marseille Cancer Research Center, Institut Paoli-Calmettes, Institut National de la Santé et de la Recherche Médicale, Centre National de la Recherche Scientifique, Aix-Marseille UniversityMarseille, France; ^3^Centre National de la Recherche Scientifique UMR 7257, Aix-Marseille UniversityMarseille, France; ^4^INRA, USC 1408 AFMBMarseille, France

**Keywords:** sheep intestinal microbiota, taxonomic diversity, carbohydrate-active enzymes (CAZymes), shotgun metagenomics, small intestine, large intestine, rectum

## Abstract

The digestive microbiota of humans and of a wide range of animals has recently become amenable to in-depth studies due to the emergence of DNA-based metagenomic techniques that do not require cultivation of gut microbes. These techniques are now commonly used to explore the feces of humans and animals under the assumption that such samples are faithful proxies for the intestinal microbiota. Sheep (*Ovis aries*) are ruminant animals particularly adapted to life in arid regions and in particular Najdi, Noaimi (Awassi), and Harrei (Harri) breeds that are raised in Saudi Arabia for milk and/or meat production. Here we report a metagenomics investigation of the distal digestive tract of one animal from each breed that (i) examines the microbiota at three intestinal subsites (small intestine, mid-colon, and rectum), (ii) performs an in-depth analysis of the carbohydrate-active enzymes genes encoded by the microbiota at the three subsites, and (iii) compares the microbiota and carbohydrate-active enzyme profile at the three subsites across the different breeds. For all animals we found that the small intestine is characterized by a lower taxonomic diversity than that of the large intestine and of the rectal samples. Mirroring this observation, we also find that the spectrum of encoded carbohydrate-active enzymes of the mid-colon and rectal sites is much richer than that of the small intestine. However, the number of encoded cellulases and xylanases in the various intestinal subsites was found to be surprisingly low, indicating that the bulk of the fiber digestion is performed upstream in the rumen, and that the carbon source for the intestinal flora is probably constituted of the rumen fungi and bacteria that pass in the intestines. In consequence we argue that ruminant feces, which are often analyzed for the search of microbial genes involved in plant cell wall degradation, are probably a poor proxy for the lignocellulolytic potential of the host.

## Introduction

The digestive tract of humans and animals is home to a diverse ecosystem of trillions of microbial cells (Qin et al., 2010; Costello et al., 2012). The digestive microbiota confers abilities that are not encoded by the mammalian genome. In particular, the intrinsic (viz. encoded by the host genome) digestive capabilities of mammals is extremely reduced and is essentially limited to starch, sucrose, and lactose (Cantarel et al., 2012; El Kaoutari et al., 2013a). One of the key roles of the digestive microbiota is to help the host digest its diet, and in particular the large array of complex carbohydrates found in the mammalian diet. The digestive microbiota of humans and of a wide range of animals has recently become amenable to in-depth studies due to the emergence of DNA-based metagenomic techniques that do not require cultivation of gut microbes. Technological progresses offer the ability to explore both taxonomical and functional profiles of the microbial DNA extracted from microbial communities with an ever-increasing depth. The most numerous microbiota investigations are certainly those of the human digestive microbiota, which has been linked to health and disease by numerous studies. The digestive microbiota of other mammals has also been studied extensively, essentially by sampling feces and subjecting them to an analysis of the taxonomical diversity via 16S RNA sequencing (Ley et al., 2008). In comparison, fewer studies have attempted to address the question of the functional digestive potential, although Muegge et al. (2011) have shown that diet shapes both the taxonomical and the functional profiles of mammalian microbiota. The major drivers of these taxonomical and functional shifts correspond to the digestive tract itself (multichamber foregut vs. hindgut fermenters) and to the actual food composition (Sanders et al., 2015). With the exception of starch, plant-derived polysaccharides are left undigested by the mammalian enzymes and are broken down and fermented by their digestive microbiota. This is particularly important for herbivorous animals that harvest their carbohydrate solely from non-starchy plant food, and especially for the species that live in harsh conditions where a maximum of energy must be derived from small amounts of plant feed. This digestion of plant derived complex carbohydrates is notoriously difficult, because of the recalcitrant nature of most plant carbohydrates that have evolved to be resistant to enzymatic digestion (Mba Medie et al., 2012) and because the variety of bonds to cleave is such that no single genome can encode the corresponding variety enzymes necessary to break plant polysaccharides to fermentable sugars (El Kaoutari et al., 2013a).

**Figure 1:**
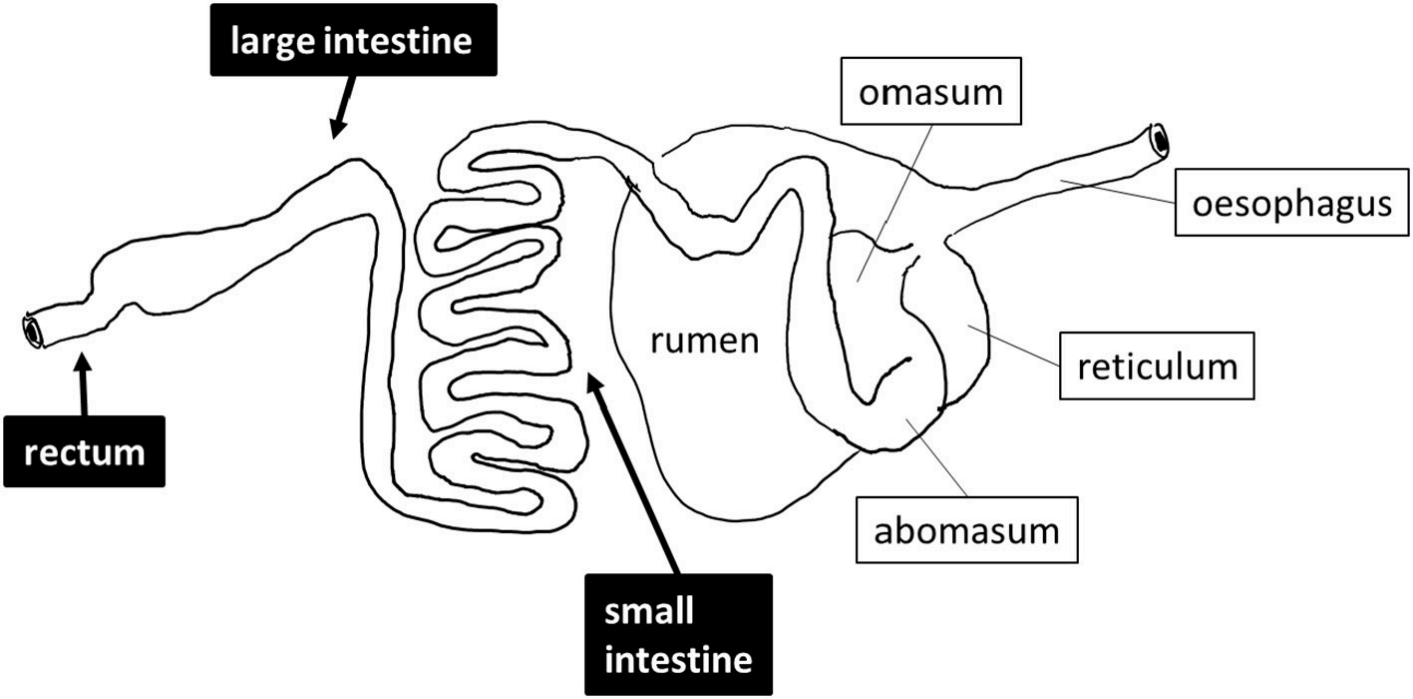
**Schematic drawing of the digestive tract of sheep**. The sites that were sampled are shown in black boxes.

Sheep (*Ovis arie*s) are exclusively herbivorous mammals that were among the earliest domesticated farm animals. Of the several hundred breeds of sheep identified by the United Nations Food and Agriculture Organization, three breeds of sheep are commonly kept as livestock in Saudi Arabia, namely Najdi, Noaimi (Awassi), and Harrei (Harri) sheep (Supplementary Figure 1). These breeds tolerate the hot and cold weather of the kingdom. In the wild these sheep graze on short green grasses, clover and hay. These three breeds are used for meat production, but Najdi and Noaimi (Awassi) sheep are also used for milk production. Like all sheep, these animals have a typical ruminant gastrointestinal tract (GIT) including four fermentation chambers, composed of the rumen, the reticulum, the omasum and the abomasum followed by a small intestine and a large intestine (Figure 1). It is widely accepted that the breakdown and fermentation of roughage is performed in the first four fermentation chambers (Herrero et al., 2013; Morgavi et al., 2013), but few studies focus on the distal gut except for feces. In order to evaluate the exact digestive role of downstream sheep intestines, we have performed a metagenomics investigation of one Najdi, one Noaimi (Awassi), and one Harrei (Harri) sheep, which examined the microbiota at three GIT subsites (small intestine, mid-colon, and rectum) for each animal. We have also performed an in-depth analysis of the carbohydrate-active enzymes genes encoded by the microbiota in order to evaluate the lignocellulolytic potential at these three subsites.

## Materials and methods

### Sample collection

Three sheep (one Najdi, one Noaimi, and one Harrei) were obtained at Jeddah's slaughterhouse market. The samples used in this study were taken immediately after animal sacrifice, following best practice veterinary care. The animals used in this study were 12–20 months old and were fed a dried mix of *Ammophila arenaria, Medicago sativa, Hordeum vulgare*, and *Sorghum bicolor* for 10–25 days at the slaughterhouse market before sacrifice. A schematic drawing of the sheep digestive system and of the sampling sites is presented in Figure 1. About 30–35 g sample from mid-small and mid-large intestine and from the rectum region were collected into sterile 50 ml Falcon tubes. The intestine samples were mostly semi liquid while the rectal samples were often solid. Immediately after collection the samples were kept refrigerated for 24–28 h then immersed in AquaStool kit solution (MultiTarget Pharmaceuticals LLC, Colorado, USA) according to the manufacturer's instructions.

### DNA extraction

Bacterial DNA was extracted and purified using the commercial QIAamp DNA Stool Mini Kit (Qiagen: https://www.qiagen.com/) according to the manufacturer's protocol. A quantity of 200 mg of each animal sample was used for DNA extraction. Quantification and assessment of extracted DNA was carried out using a NanoDrop ND-1000 spectrophotometer (NanoDrop, Inc.). The extracted materials were stored at −80°C until use. The concentration of the extracted DNA is reported in Supplementary Table 1.

### Metagenomics analyses

The samples were submitted to LGC Genomics GmbH (Berlin, Germany) for all sequencing tasks. The sequencing technology chosen was Illumina MiSeq V3 delivering 300 bp paired-end reads. All sequence reads were deposited in the Sequence Read Archive (Leinonen et al., 2011).

### 16S rDNA amplicon sequencing and data pre-processing

The V3–V4 regions of the bacterial 16S rRNA gene were amplified using two universal primers 341F (CCTACGGGNGGCWGCAG) and 785R (GACTACHVGGGTATCTAAKCC) complemented with sample-specific barcodes for multiplexing during sequencing. The primary sequencing output data were demultiplexed using the Illumina bcl2fastq 1.8.4 tool to generate the FASTQ files corresponding to each library. Before clipping the barcode and the adapter sequences, reads with the following criteria were filtered out: (i) reads with more than one mismatch per barcode, (ii) reads with missing barcodes, one-sided barcodes, or conflicting barcode pairs and (iii) reads with a final length <100 bases.

Additional filtering criteria were considered before primer detection and clipping. Thus, only reads with pairs of primers (Fw-Rev or Rev-Fw) and reads with less than three mismatches per primer were kept for further analysis. Furthermore, all reads were turned into forward-reverse primer orientation after removing the primer sequences. The forward and reverse reads were combined using BBMerge v34.48 (http://bbmap.sourceforge.net/). Read quality was controlled using FastQC (http://www.bioinformatics.babraham.ac.uk/projects/fastqc/) for each sample. Reads with an average Phred quality score <33 or containing ambiguous bases were removed.

Operational Taxonomic Units (OTU) clustering has been performed using Mothur v1.35.1 using a 97% identity threshold (cluster.split method). The representative sequences of each OTU were queried against the Ribosomal Database Project release 11.4 (https://rdp.cme.msu.edu/) using BLAST with the following parameters: *E*-value of 1 or less and percent identity of 90% or higher. Next, the OTU diversity was analyzed using QIIME v1.9.0 (http://qiime.org/) following the standard workflow to determine the membership of all OTUs to different taxonomic levels and groups. The within-sample (alpha diversity and Shannon's diversity index H) as well as between-sample diversity (beta) were calculated using the R package “vegan” (Oksanen et al., 2017). Downstream analysis and result visualization were done with custom R scripts based on the “ggplot2” package (Wickham, 2009). Hierarchical clustering between samples was performed using “hclust” function of R (R Core Team, 2016) with “complete” method applied to a distance matrix derived from the beta diversity using the “betadiver” function from the “vegan” package.

### Metagenomic shotgun sequence data analysis

The pre-processing of the shotgun sequencing output data was performed following the same workflow as described above. Assembly of the reads was performed using Trinity (Grabherr et al., 2011) with default parameters. Supplementary Table 2 reports the sequencing and assembly statistics for each sample.

### Carbohydrate-active enzymes analysis from metagenomics data

The detection of the assembled DNA sequences that encode carbohydrate-active enzymes was done using FASTY (Pearson et al., 1997) against a custom sequence library made of the catalytic domains of ~150,000 sequences of glycoside hydrolase (GH), polysaccharide lyase (PL), glycosyl transferases (GT), carbohydrate esterases (CE), auxiliary activity (AA), and of their associated carbohydrate-binding modules (CBMs) derived from the carbohydrate-active enzymes database (www.cazy.org; Lombard et al., 2014). We retained as positive hits those that had 30% identity or more and an *e*-value of 10^−6^ or better with a sequence in the library.

## Results and discussion

### Community structure and diversity derived from 16S analysis

A total of 625,937 16S rRNA sequences were obtained from the three intestinal sites sampled (small intestine, large intestine, and rectum) of three sheep. The obtained 16S rRNA sequences represented 7,147 unique OTUs which could be assigned to 24 phyla, 69 order, and 186 genera. Overall, the bacterial communities were mostly dominated by *Firmicutes* (42.5% of the total sequences and 72% of different OTUs) followed by *Bacteroidetes* (21.5% sequences and 13.9% OTUs), *Proteobacteria* (11.5% of total sequences and 2.6% of OTUs), Candidate Division *TM7* (7.7% of sequences and 3.4% of OTUs), *Actinobacteria* (2.5% of total sequences and 2.8% of OTUs), and *Planctomycetes* (6% of sequences and 1.5% of OTUs). Other phyla were represented in minor proportions ranging from 2% to <0.1% of sequences and OTUs (Table 1).

**Table 1:** **Abundance (number of sequences) and diversity (OTUs) at the phylum level for all sheep samples (combined)**.

**Phylum**	**Total sequences**	**%**	**Unique OTUs**	**%**
*Acidobacteria*	4	0.0	2	0.03
*Actinobacteria*	7,112	2.2	133	1.9
*Bacteroidetes*	61,962	19.4	817	11.4
*BD1–5*	2	0.0	1	0.01
*Candidate_division_OD1*	28	0.01	5	0.05
*Candidate_division_TM7*	21,457	6.7	130	1.8
*Chlamydiae*	49	0.01	3	0.05
*Chloroflexi*	4	0.0	4	0.06
*Cyanobacteria*	3,796	1.2	178	2.5
*Deferribacteres*	39	0.01	3	0.05
*Deinococcus-Thermus*	12	0.0	1	0.02
*Elusimicrobia*	243	0.1	10	0.1
*Fibrobacteres*	644	0.2	11	0.2
*Firmicutes*	135,597	42.5	4,018	56.2
*Fusobacteria*	109	0.05	5	0.1
*Lentisphaerae*	5,293	1.7	140	2.0
*Nitrospirae*	1	0.0	1	0.01
*Planctomycetes*	16,945	5.3	61	0.9
*Proteobacteria*	34,077	10.7	182	2.6
*SHA-109*	39	0.01	4	0.05
*Spirochaetae*	2,271	0.7	58	0.8
*Synergistetes*	30	0.01	4	0.05
*Tenericutes*	6,679	2.1	240	3.4
Unclassified	16,735	5.2	1,085	15.2
*Verrucomicrobia*	5,811	1.8	51	0.7

The taxonomic profiles were globally similar between the three animals along the intestinal tract especially at the phylum level. The core intestinal tract microbiota of the three sheep was composed of the major phyla including *Firmicutes, Bacteroidetes, Actinobacteria*, Candidate division *TM7* and *Proteobacteria*.

The large intestine and rectal microbiota profiles of the Najdi sheep were largely similar despite minor taxonomic differences (Supplementary Figure 2). In fact, the rectal microbiota of this animal was characterized by higher number of OTUs within *Firmicutes* especially *Clostridiales* order with 1,152 different OTUs vs. 845 OTUs in large intestine. *Bacteroidales* (main order of *Bacteroidetes*) were similarly represented in both large intestine and rectum while this phylum was more abundant in the large intestine (33.7% of sequences) than in the rectum (17.6% of sequences). The diversity within the three GIT subsites in the three animals is given in Supplementary Figure 4. The small intestine of Najdi sheep had a unique taxonomic profile compared to the large intestine and rectum, with a very low diversity (α-diversity index 39.5) and a particularly high number of sequences that belong to *Proteobacteria* with 66% of total sequences corresponding to *Escherichia* and *Shigella* genera. *Lactobacillus* genus (*Firmicutes*) was also highly represented in small intestine of Najdi sheep with 29% of total sequences (Supplementary Table 3).

Samples derived from the large intestine and rectum of the Noaimi sheep had very similar taxonomic profiles at both phylum and order level. They were characterized by the predominance of *Firmicutes* (mainly *Clostridiales*), *Bacteroidetes* (mainly *Bacteroidales*) and *Planctomycetes* (mainly *Planctomycetales*). The number of sequences and OTUs at the Order level are given in Supplementary Table 4.

**Figure 2:**
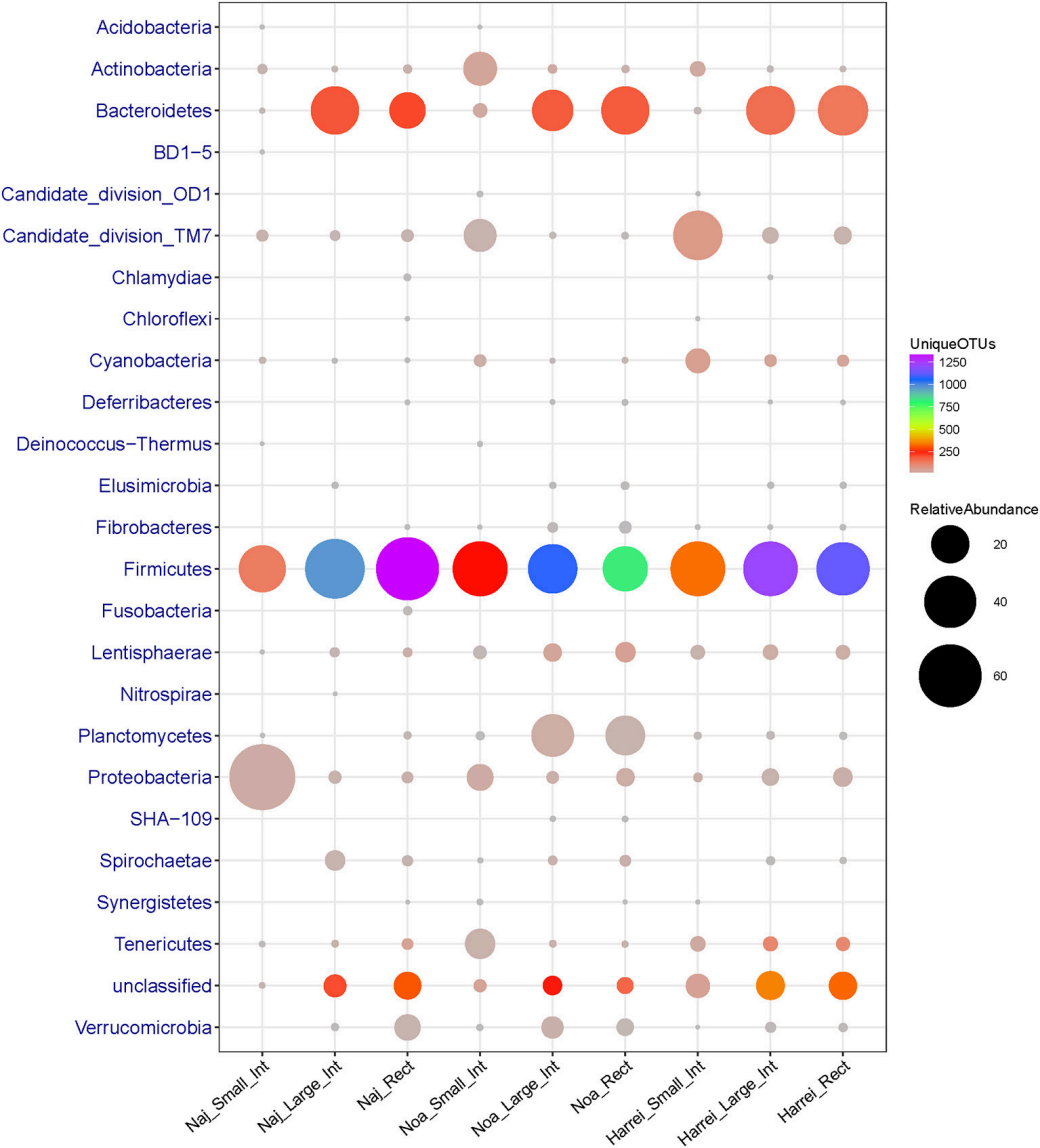
**Abundance (number of 16S rRNA sequences) and diversity (number of unique OTUs) across the three sheep and the various intestinal subsites at the phylum level**. The size of the circles is proportional to the total number of sequences and the color represents the number of unique OTUs for each given taxonomic group and sample.

Here again the small intestine had a distinct taxonomic profile compared to other intestinal subsites. It was characterized by a low diversity of different bacterial groups (only 555 OTUs in total and α-diversity index 92.1) and a particularly low relative abundance of *Bacteroidetes* members which represent <1.5% of sequences in total. In addition, the small intestine was enriched in other phyla such as *Actinobacteria, Tenericutes, Proteobacteria*, and Candidate division *TM7* (new candidate phylum) compared to the other subsites (Figure 2 and Table 2).

**Table 2:** **Abundance (number of 16S rRNA sequences) and diversity (number of unique OTUs) across the three sheep and the various intestinal subsites at the phylum level**.

	**Najdi sheep**						**Noiami sheep**						**Harrei sheep**					
	**Small intestine**		**Large intestine**		**Rectum**		**Small intestine**		**Large intestine**		**Rectum**		**Small intestine**		**Large intestine**		**Rectum**	
**Phylum**	**Seq**	**OTUs**	**Seq**	**OTUs**	**Seq**	**OTUs**	**Seq**	**OTUs**	**Seq**	**OTUs**	**Seq**	**OTUs**	**Seq**	**OTUs**	**Seq**	**OTUs**	**Seq**	**OTUs**
*Acidobacteria*	3	1	0	0	0	0	1	1	0	0	0	0	0	0	0	0	0	0
*Actinobacteria*	205	18	20	11	116	25	5,745	52	126	38	75	24	775	40	31	11	19	12
*Bacteroidetes*	13	11	11,218	200	5,882	216	601	40	7,908	193	12,386	192	53	13	11,202	162	12,699	145
*BD1–5*	2	1	0	0	0	0	0	0	0	0	0	0	0	0	0	0	0	0
*Candidate division OD1*	0	0	0	0	0	0	24	2	0	0	0	0	4	3	0	0	0	0
*Candidate_division_TM7*	366	23	191	14	359	22	5,364	23	41	8	54	7	13,253	77	766	21	1,063	22
*Chlamydiae*	0	0	0	0	45	3	0	0	0	0	0	0	0	0	4	1	0	0
*Chloroflexi*	0	0	0	0	2	2	0	0	0	0	0	0	2	2	0	0	0	0
*Cyanobacteria*	42	19	10	4	5	5	400	33	11	7	26	12	2,620	63	343	53	339	52
*Deferribacteres*	0	0	0	0	4	2	0	0	6	2	23	2	0	0	2	1	4	1
*Deinococcus-Thermus*	1	1	0	0	0	0	11	1	0	0	0	0	0	0	0	0	0	0
*Elusimicrobia*	0	0	38	3	0	0	0	0	39	3	92	5	0	0	36	3	38	3
*Fibrobacteres*	0	0	0	0	6	1	3	2	208	5	394	3	8	1	7	1	18	2
*Firmicutes*	12,630	136	17,807	965	19,953	1,316	17,089	266	11,754	1,067	10,720	817	16,665	332	14,461	1,212	14,518	1,125
*Fusobacteria*	0	0	0	0	109	5	0	0	0	0	0	0	0	0	0	0	0	0
*Lentisphaerae*	2	2	153	17	139	30	535	10	1,033	50	1,585	60	631	21	636	32	579	31
*Nitrospirae*	0	0	1	1	0	0	0	0	0	0	0	0	0	0	0	0	0	0
*Planctomycetes*	4	4	0	0	75	10	129	1	8,466	33	8,037	22	67	6	96	7	71	2
*Proteobacteria*	26,333	36	382	24	284	27	3,170	37	362	30	1,231	33	157	29	884	23	1,274	29
*SHA-109*	0	0	0	0	0	0	0	0	17	3	22	3	0	0	0	0	0	0
*Spirochaetae*	0	0	1,411	20	241	18	17	2	149	22	300	25	0	0	110	4	43	4
*Synergistetes*	0	0	0	0	1	1	25	2	0	0	2	1	2	1	0	0	0	0
*Tenericutes*	22	5	53	23	274	57	4,419	26	54	24	35	21	725	40	579	113	518	110
Unclassified	26	16	1,885	213	2,975	306	462	56	1,234	256	946	173	2,481	58	3,412	354	3,314	322
*Verrucomicrobia*	0	0	72	4	2,623	21	39	1	1,724	25	1,018	12	1	1	212	7	122	7

The microbial communities within the intestinal tract of the Harrei sheep followed the same trend as with the two other sheep with the highest microbial diversity residing in the large intestine and rectum, and a very low abundance of *Bacteroidetes* in the small intestine. We observed the highest abundance of Candidate division *TM7* and *Cyanobacteria* in the small intestine compared to the two other subsites (Figure 2 and Table 2).

The distribution of microbial communities at a given intestinal subsite thus appears to be stable across the three animals studied here. The microbial diversity and the relative abundance were correlated to the body subsite. Overall, the small intestine was characterized by low microbial diversity and abundance compared to the large intestine and rectum (Supplementary Figure 4). At the phylum level, the most striking observation is the very low abundance of *Bacteroidetes* in the small intestine of the three sheep compared to the other intestinal subsites. The large intestine and rectal bacterial communities were characterized by a high predominance of members of *Firmicutes* (*Clostridiales*) and *Bacteroidetes* (*Bacteroidales*).

Hierarchical clustering based on beta diversity between different sheep and intestinal subsites confirmed the results described above. Supplementary Figure 3 shows that sheep microbiota diversity in large intestine and rectum were similar to each other (especially within the same animal) while all samples from small intestine showed a particular profile and clustered together.

Finally we note that a large portion of identified OTUs from different samples did not belong to any known or candidate phylum and was flagged as “unclassified.” The proportion of unclassified OTUs varied between ~8% in the small intestine to ~18% in the large intestine of Harrei sheep (Figure 2 and Table 2).

### CAZyme analysis (functional analysis)

The reads of each sample were assembled into contigs. While the final number of reads obtained for each sample was of the same order of magnitude (between 1.5 and 2.9 million reads), the number of assembled contigs was lower and with a larger average length for the three small intestine samples (Supplementary Table 2), suggesting that the small intestine of sheep may be characterized by a lower organismal concentration than the other two subsites. By contrast there was no major difference in the number of assembled contigs and in the contig length for the large intestine or rectal samples.

**Figure 3:**
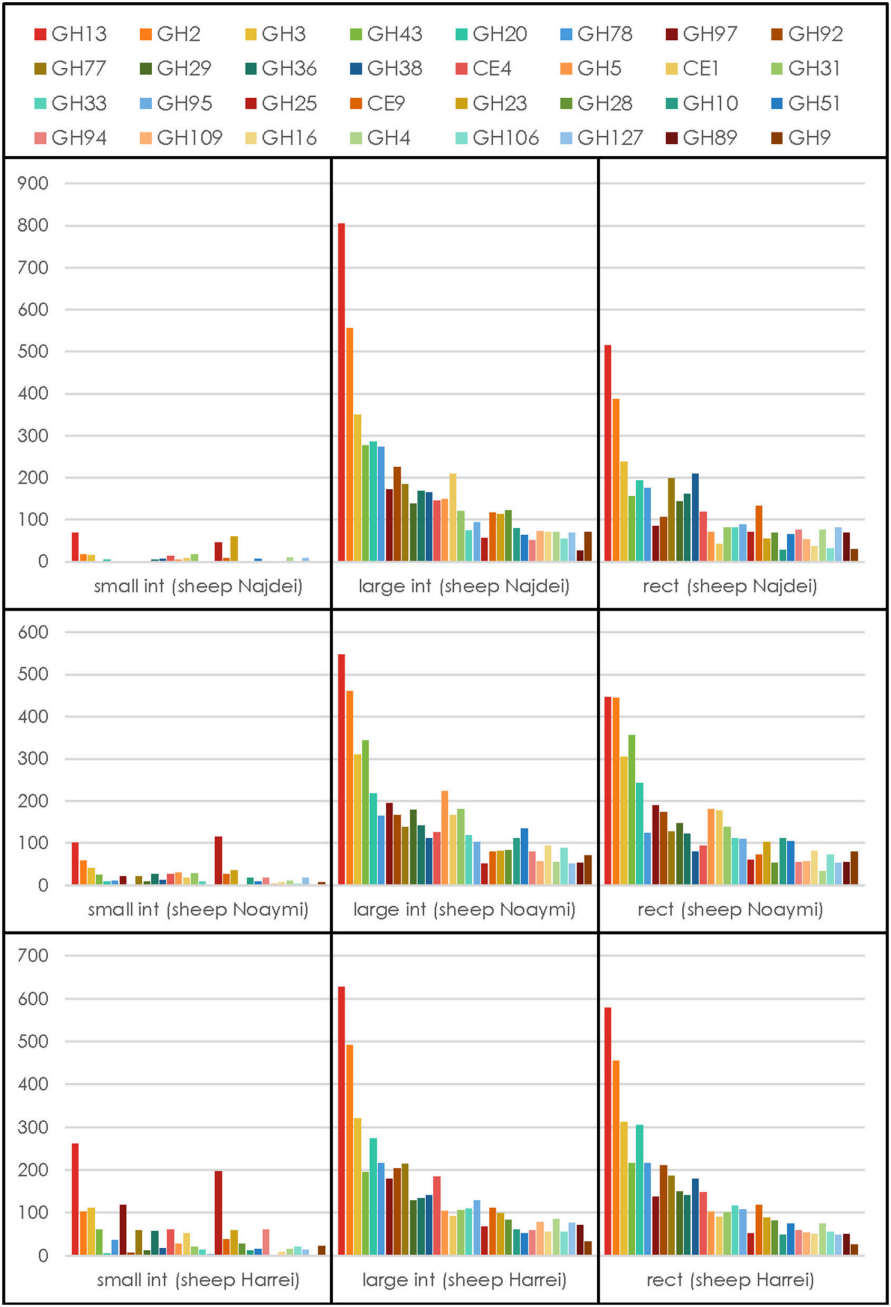
**CAZymes profile for each animal at each subsite**. The average number for each GH or PL family across subsites and animals was used to select the 32 most abundant degradative families.

Because carbohydrate-active enzymes often have a modular structure where the catalytic domain is appended to a variable number of other domains that may be catalytic or carbohydrate-binding, all contigs were analyzed by pairwise alignment to a library of ~150,000 individual functional domains (modules) derived from the five main categories of modules described in the carbohydrate-active enzymes database, namely glycoside hydrolases (GH), polysaccharide lyases (PL), carbohydrate esterases (CE), glycosyltransferases (GT), auxiliary activity (AA), and CBMs (Lombard et al., 2014). From a total number of ~1.5 million contigs, 59,337 genes encoding carbohydrate-active enzymes were detected (average ~6,600 per body site), representing ~4% of the total of contigs that were assembled. The count of CAZy families for each body site and each animal is given in Supplementary Table 5. No similarity to enzymes of the AA category could be detected, a finding not unexpected given that AA enzymes are oxygen-utilizing oxidoreductases and the three samples subsites are strictly anaerobic. The relative abundance of the families of CAZymes involved in glycosidic bond cleavage [the glycoside hydrolases (GHs) and polysaccharide lyases (PLs)] was computed and the resulting profiles are shown in Figure 3 for the 32 most abundant families. Regardless of the animal, the profile of the GHs and PLs of the small intestine samples is much lower than that in the other sites likely due to the lower concentration of bacteria, a situation similar to that encountered in the human duodenum, where only a very limited number of CAZymes can be found due to the lower concentration of resident bacteria (Angelakis et al., 2015). On the other hand, when the 32 most abundant families were ranked by their average abundance across subsites, the same families tended to be found at the same rank, regardless of the animal (Figure 3) for the large intestine and rectal subsites. By contrast the family profile of the three small intestine samples was markedly different, with a much lower average value and different rank for the most abundant families. This observation mirrors what was observed upon 16S analysis, with the large intestine and rectal subsites being much richer and diverse than the small intestine.

We have then examined the putative function encoded by the most abundant carbohydrate-active enzymes families in the large intestine and rectal samples, viz. the sites with the most abundant profiles (Figure 3). In these sites, regardless of the animal and for both sites, GH13 is consistently the most abundant family, reflecting the universal role of starch/glycogen as a central nutrient source and reserve macromolecule. The other most abundant families include families with miscellaneous substrates (GH2 and GH3), and more specific families like GH43 (α-L-arabinofuranosidases), GH20 (N-acetyl β-glucosaminidases), GH78 (α-L-rhamnosidases), GH97 (starch), GH92 and GH38 (α-mannosidases), GH77 (amylomaltase), GH29 and GH95 (α-L-fucosidases), GH36 (α-galactosidases), CE4 and CE1 (deacetylases), GH33 (sialidases and other ulosonic acid hydrolases), GH25 (lysozymes). Interestingly, the relative abundance of enzymes that target cellulose and xylan is lower than what was found in a large scale metagenomics investigation of the cow rumen (Hess et al., 2011; Table 3).

**Table 3:** **Number of cellulases and xylanases in cow rumen and in sheep; α-mannosidase and β-N-acetylglucosaminidases are shown for comparison**.

**Activity**	**Family**	**Cow rumen^§^**	Sheep^*^
Cellulase	GH9	795	222
	GH45	115	6
	GH48	3	1
Xylanase	GH10	1,025	316
	GH11	165	5
α-mannosidase	GH38	272	635
β-N-acetylglucosaminidase	GH20	765	1,088

§ *Taken from Hess et al. (2011)*.

* *This work; average of large intestine and rectum for the three animals, scaled to the same overall number of GHs as in Hess et al. (2011)*.

## Conclusions

Most studies of the digestive microbiota focus on animal (or human) feces, which are assumed to be representing the diversity (taxonomical and functional) of the microbes involved in the breakdown of complex carbohydrates and glycans. Fewer studies focus on the rumen of herbivores and even less on the intestines. Our study reveals a remarkable functional stability of the CAZyme profiles across the large intestine and rectal sites of all examined sheep. This functional resilience is in agreement with earlier observations made on the human gut microbiota (El Kaoutari et al., 2013b). The functional redundancy in the digestive microbiota ruminants can explain the adaptability of these animals toward various types of feeds and to rapid dietary changes.

The families of enzymes that target cellulose and xylan, were found to be less abundant in the sheep distal gut sites than in metagenomics studies of the cow rumen. Inversely, families of enzymes involved in peptidoglycan (for instance GH25) and fungal polysaccharides (for instance GH16, GH20) were found to be more abundant than in the rumen. Taken together these observations strengthen the idea that the bulk of cellulose breakdown is performed in the upperpart of the gastrointestinal tract (i.e., in the rumen) and that the carbon sources of the distal intestinal flora may comprise fungal and bacterial cells that have passed from the rumen into the intestines, along with residual fibers. An unexpected consequence is the realization that ruminant feces, which are often analyzed for the search of microbial genes involved in plant cell wall degradation, are probably a poor proxy for the lignocellulolytic potential of their host.

## Ethics statement

The animals were purchased at the slaughter house of Jeddah, Saudi Arabia, immediately after sacrifice.

## Authors contributions

Conceptualization, BH, SA, ER, and HA; Sample collection, SA; DNA extraction, AE; Computer analyses of carbohydrate-active enzymes, ED and VL; Data analysis, BH, AE, VL; Writing, BH with help from AE, SA, and VL.

### Conflict of interest statement

The handling Editor declared a shared affiliation, though no other collaboration, with several of the authors ED, VL, BH and states that the process nevertheless met the standards of a fair and objective review. The other authors declare that the research was conducted in the absence of any commercial or financial relationships that could be construed as a potential conflict of interest.

## Supplementary material

Supplementary Figure 1 **Photos of the three breeds of sheep that were studied. (A)** Najdi; **(B)** Noaimi; **(C)** Harrei.

Supplementary Figure 2 **Relative abundance (% of 16S rRNA sequences) and diversity (number of unique OTUs) across the three sheep and the various intestinal subsites at the order level**. The size of the circles is proportional to the % of sequences and the color represents the number of unique OTUs for each given taxonomic group and sample.

Supplementary Figure 3 **Hierarchical clustering of samples based on beta diversity**.

Supplementary Figure 4 **Scatterplot of Shannon's diversity index (H) and Fisher's alpha diversity index for all samples**.

Supplementary Table 1 **DNA concentration for each extracted subsite**.

Supplementary Table 2 **Sequencing and assembly statistics for metagenomics data obtained with each sample**.

Supplementary Table 3 **Abundance (number of 16S rRNA sequences) and diversity (number of unique OTUs) across the three sheep and the various intestinal subsites at the genus level**.

Supplementary Table 4 **Abundance (number of 16S rRNA sequences) and diversity (number of unique OTUs) across the three sheep and the various intestinal subsites at the phylum level**.

Supplementary Table 5 **Number of CAZymes detected by family, for each animal and each subsite**.
