# Supplementary material for: A Metagenomics Investigation of Carbohydrate-Active Enzymes along the Gastrointestinal Tract of Saudi Sheep
Source: Front Microbiol. 2017 Apr 20;8:666. doi: 10.3389/fmicb.2017.00666 (PMC5397404; doi:10.3389/fmicb.2017.00666)
Supplement: Supplementary Figure 1 — Photos of the three breeds of sheep that were studied. (A) Najdi; (B) Noaimi; (C) Harrei. [file Image1.PDF]

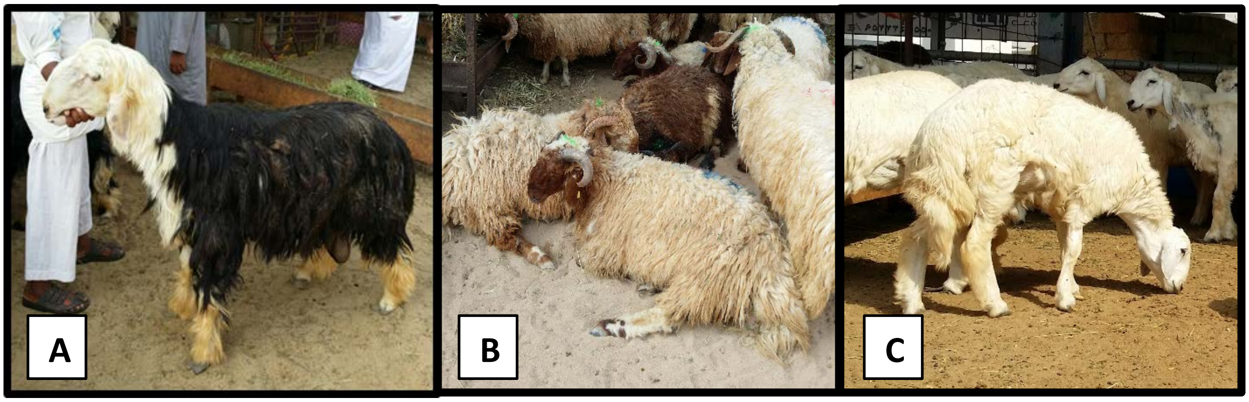

Supplementary Figure 1: photos of the three breeds of sheep that were studied. A, Najdi; B, Noaimi; C, Harrei
