# Supplementary figures and images for: A Metagenomics Investigation of Carbohydrate-Active Enzymes along the Gastrointestinal Tract of Saudi Sheep
Source: Front Microbiol. 2017 Apr 20;8:666. doi: 10.3389/fmicb.2017.00666 (PMC5397404; doi:10.3389/fmicb.2017.00666)

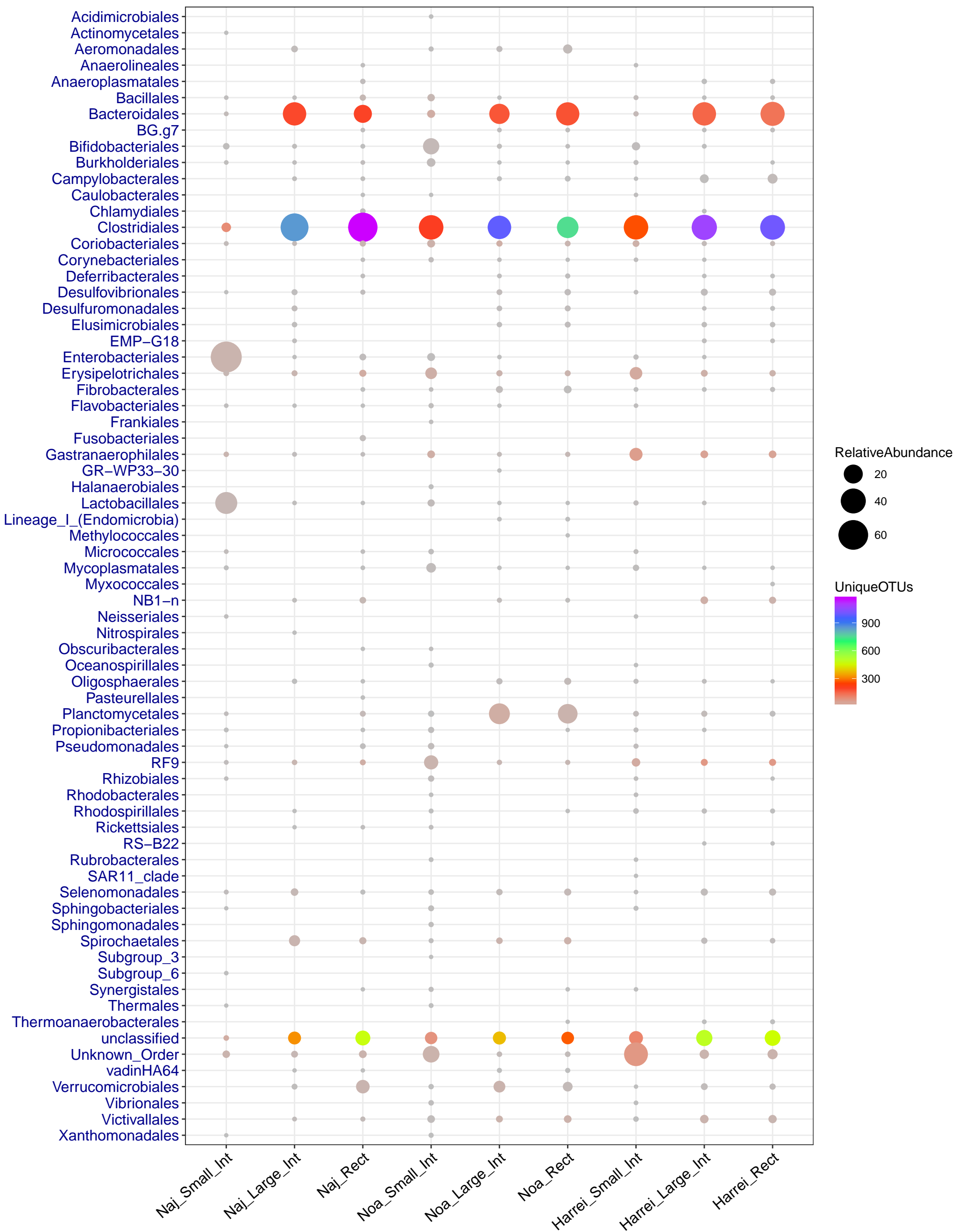

Supplement: Supplementary Figure 2 — Relative abundance (% of 16S rRNA sequences) and diversity (number of unique OTUs) across the three sheep and the various intestinal subsites at the order level. The size of the circles is proportional to the % of sequences and the color represents the number of unique OTUs for each given taxonomic group and sample. [file Image2.PDF]

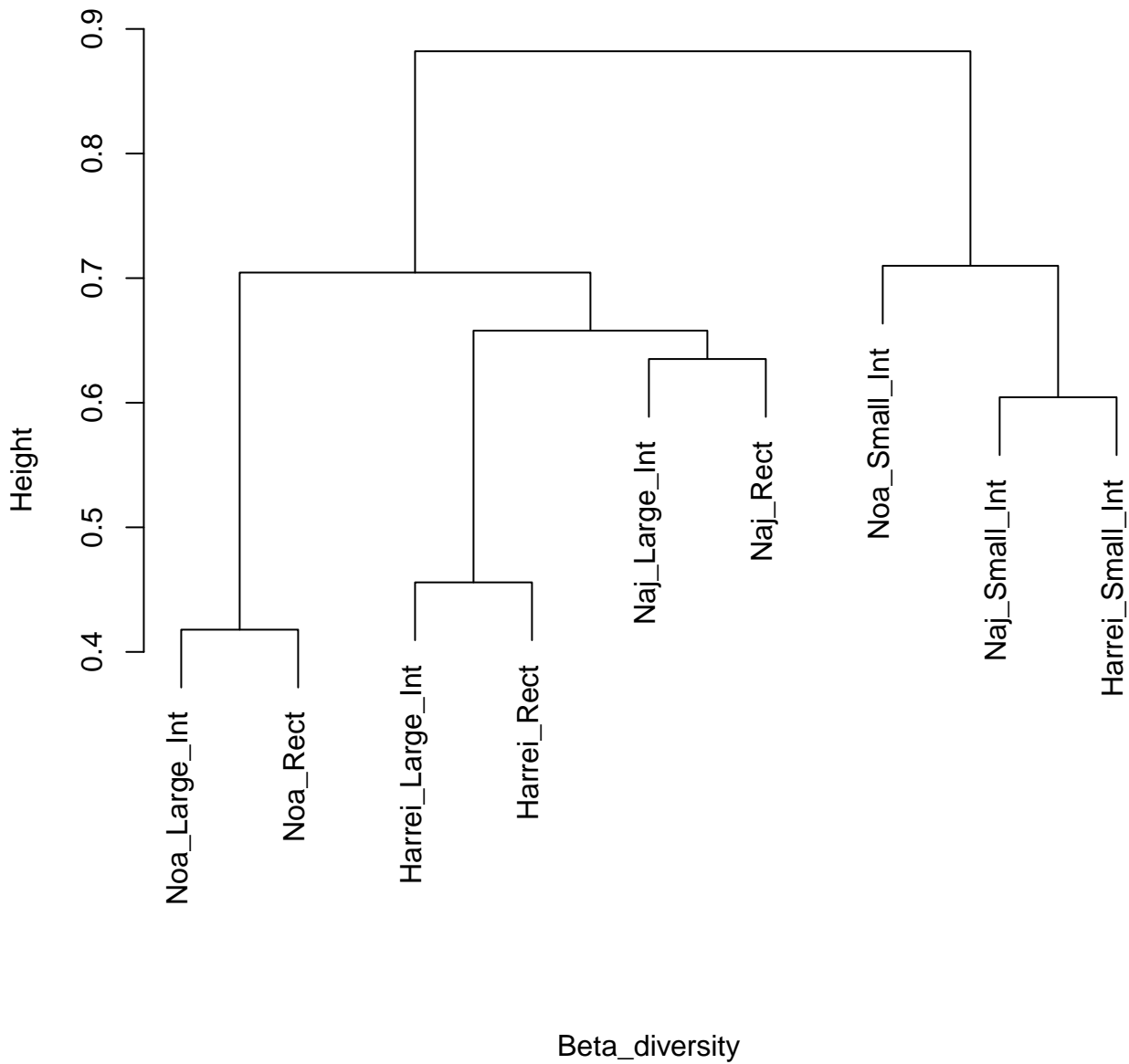

Supplement: Supplementary Figure 3 — Hierarchical clustering of samples based on beta diversity. [file Image3.PDF]

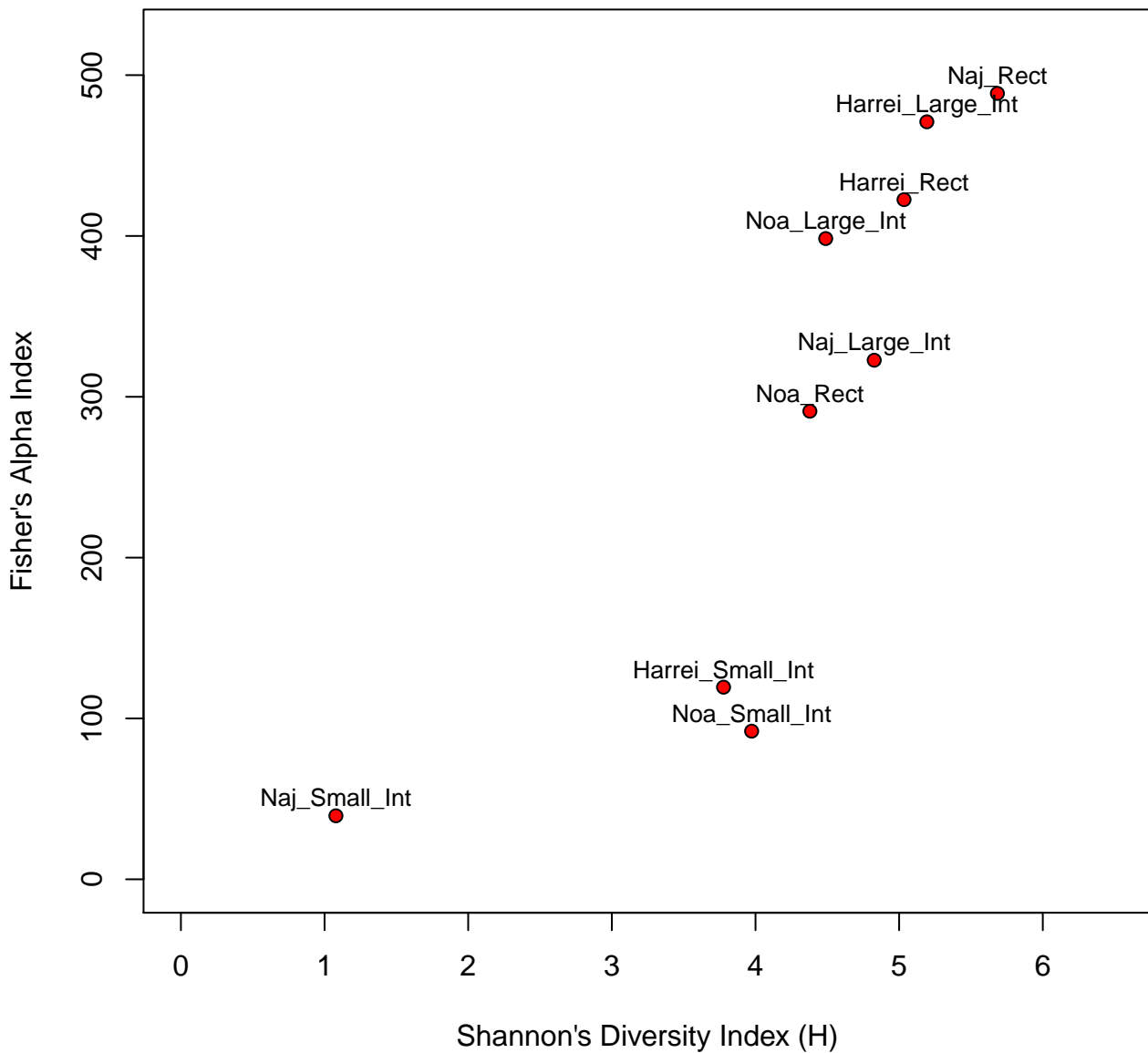

Supplement: Supplementary Figure 4 — Scatterplot of Shannon's diversity index (H) and Fisher's alpha diversity index for all samples. [file Image4.PDF]
