# Supplementary material for: A Metagenomics Investigation of Carbohydrate-Active Enzymes along the Gastrointestinal Tract of Saudi Sheep
Source: Front Microbiol. 2017 Apr 20;8:666. doi: 10.3389/fmicb.2017.00666 (PMC5397404; doi:10.3389/fmicb.2017.00666)
Supplement: Supplementary Table 1 — DNA concentration for each extracted subsite. [file Table1.PDF]

Supplementary Table 1. DNA concentration for each extracted subsite

**Animal**	**Body site**	**DNA extracted (ng/µl)**
Sheep Najdei	Small intestine	3.8
	Large intestine	60.4
	Rectum	7.3
Sheep Noaimi	Small intestine	2.9
	Large intestine	10.2
	Rectum	17.3
Sheep Harrei	Small intestine	2.2
	Large intestine	10.9
	Rectum	13.9
