# Supplementary material for: A Metagenomics Investigation of Carbohydrate-Active Enzymes along the Gastrointestinal Tract of Saudi Sheep
Source: Front Microbiol. 2017 Apr 20;8:666. doi: 10.3389/fmicb.2017.00666 (PMC5397404; doi:10.3389/fmicb.2017.00666)
Supplement: Supplementary Table 2 — Sequencing and assembly statistics for metagenomics data obtained with each sample. [file Table2.PDF]

Supplementary Table 2. Sequencing and assembly statistics for metagenomics data obtained with each sample

sample source	number of raw sequences	number of contigs	contig average length (bases)
small intestine (sheep Najdei)	1978939	18542	1983
large intestine (sheep Najdei)	1743631	213500	826
rectum (sheep Najdei)	1622509	231872	506
small intestine (sheep Noaimi)	2883640	62148	1005
large intestine (sheep Noaimi)	1891499	224910	792
rectum (sheep Noaimi)	1751650	199047	814
small intestine (sheep Harrei)	1543223	97324	1334
large intestine (sheep Harrei)	1794513	240023	687
rectum (sheep Harrei)	1751770	229202	687
