# Supplementary material for: A Metagenomics Investigation of Carbohydrate-Active Enzymes along the Gastrointestinal Tract of Saudi Sheep
Source: Front Microbiol. 2017 Apr 20;8:666. doi: 10.3389/fmicb.2017.00666 (PMC5397404; doi:10.3389/fmicb.2017.00666)
Supplement: Supplementary Table 3 — Abundance (number of 16S rRNA sequences) and diversity (number of unique OTUs) across the three sheep and the various intestinal subsites at the genus level. [file Table3.PDF]

Supplementary Table 3. Abundance (number of 16S rRNA sequences) and diversity (number of unique OTUs) across the three sheep and the various intestinal subsites at the genus level

	Najdi sheep						Noiami sheep						Harrei sheep					
	Small intestine		Large intestine		Rectum		Small intestine		Large intestine		Rectum		Small intestine		Large intestine		Rectum	
**Genus**	Seq	OTUs	Seq	OTUs	Seq	OTUs	Seq	OTUs	Seq	OTUs	Seq	OTUs	Sequ	OTUs	Seq	OTUs	Seq	OTUs
Acetitomaculum	18	7	18	4	13	6	424	10	7	3	2	2	328	16	2	1	4	3
Acidaminococcus	0	0	0	0	0	0	8	1	0	0	0	0	0	0	0	0	0	0
Acidovorax	0	0	0	0	0	0	9	1	0	0	0	0	0	0	0	0	0	0
Acinetobacter	0	0	0	0	0	0	8	1	0	0	0	0	0	0	0	0	0	0
Actinobacillus	0	0	0	0	2	1	0	0	0	0	0	0	0	0	0	0	0	0
Actinobaculum	1	1	0	0	0	0	0	0	0	0	0	0	0	0	0	0	0	0
Aeriscardovia	2	1	9	2	8	1	5056	10	15	2	4	2	537	4	8	1	0	0
Akkermansia	0	0	71	3	2622	20	39	1	1703	21	992	9	1	1	190	5	112	5
Alistipes	0	0	2978	19	812	28	0	0	237	12	396	10	0	0	4144	24	5879	23
Allisonella	2	1	0	0	0	0	0	0	0	0	0	0	0	0	0	0	0	0
Anaerofilum	0	0	21	2	15	1	0	0	2	1	2	1	0	0	18	1	18	1
Anaerofustis	1	1	0	0	16	7	14	2	47	5	30	4	140	5	11	5	13	3
Anaeroplasma	0	0	0	0	34	4	0	0	0	0	0	0	0	0	34	1	27	2
Anaerosporobacter	0	0	14	3	0	0	0	0	5	2	9	1	0	0	16	2	13	2
Anaerostipes	0	0	0	0	1	1	0	0	0	0	0	0	0	0	1	1	3	1
Anaerotruncus	5	2	92	19	134	22	46	2	44	14	54	19	1	1	120	20	138	23
Anaerovibrio	1	1	6	2	0	0	0	0	1	1	2	2	0	0	0	0	0	0
Anaerovorax	0	0	9	2	8	2	141	3	27	8	23	5	24	2	55	3	37	2
Anoxybacillus	0	0	0	0	0	0	6	1	0	0	0	0	2	1	0	0	0	0
Atopobium	5	2	2	2	8	5	129	5	7	3	5	1	160	9	6	2	7	3
Bacillus	0	0	1	1	24	4	49	2	0	0	0	0	0	0	2	1	4	2
Bacteroides	0	0	1490	22	252	20	108	4	818	20	1591	26	3	1	1094	24	1277	26
Barnesiella	0	0	14	2	59	3	0	0	3	1	19	1	0	0	104	6	71	3
Bifidobacterium	174	2	2	1	0	0	0	0	0	0	0	0	0	0	0	0	0	0
Blautia	2	2	68	5	28	5	13	1	229	4	232	4	25	3	181	5	93	5
Blvii28_wastewater-sludge_group	0	0	0	0	0	0	8	1	0	0	0	0	0	0	0	0	0	0
Brachybacterium	0	0	0	0	0	0	0	0	0	0	0	0	8	1	0	0	0	0
Bradyrhizobium	1	1	0	0	0	0	101	1	0	0	0	0	2	1	0	0	1	1
Brevundimonas	0	0	0	0	1	1	1	1	0	0	0	0	1	1	0	0	0	0

Brochothrix	1	1	0	0	0	0	44	2	1	1	0	0	6	1	0	0	0	0
Butyricicoccus	0	0	50	3	1	1	0	0	17	2	20	1	0	0	39	1	27	1
Butyricimonas	0	0	0	0	0	0	0	0	0	0	0	0	0	0	16	5	14	6
Butyrivibrio	12	2	5	3	15	5	63	4	2	1	2	2	245	12	7	4	4	2
Caldicoprobacter	0	0	0	0	0	0	0	0	0	0	2	1	0	0	1	1	0	0
Campylobacter	0	0	7	2	3	2	0	0	13	1	71	1	0	0	615	3	979	7
Candidatus_Captivus	0	0	0	0	0	0	1	1	0	0	0	0	0	0	0	0	0	0
Candidatus_Hepatincola	0	0	1	1	1	1	0	0	0	0	0	0	0	0	0	0	0	0
Candidatus_Pelagibacter	0	0	0	0	0	0	0	0	0	0	0	0	1	1	0	0	0	0
Candidatus_Saccharimonas	363	21	191	14	359	22	5364	23	41	8	54	7	13247	74	766	21	1063	22
Candidatus_Solibacter	0	0	0	0	0	0	1	1	0	0	0	0	0	0	0	0	0	0
Cellulosilyticum	0	0	11	1	7	1	16	1	0	0	0	0	5	3	0	0	1	1
Cetobacterium	0	0	0	0	106	2	0	0	0	0	0	0	0	0	0	0	0	0
Chlamydia	0	0	0	0	36	1	0	0	0	0	0	0	0	0	0	0	0	0
Chlamydophila	0	0	0	0	6	1	0	0	0	0	0	0	0	0	4	1	0	0
Christensenella	0	0	1	1	36	4	26	1	48	8	46	5	1	1	10	6	7	5
Chryseobacterium	2	1	0	0	0	0	0	0	1	1	0	0	0	0	0	0	0	0
Citricoccus	1	1	0	0	0	0	0	0	0	0	0	0	0	0	0	0	0	0
Cloacibacillus	0	0	0	0	1	1	0	0	0	0	2	1	0	0	0	0	0	0
Clostridium_sensu_stricto_1	359	3	100	3	43	1	342	2	4	2	18	4	146	3	13	2	1	1
Clostridium_sensu_stricto_11	0	0	0	0	0	0	32	1	0	0	0	0	0	0	0	0	0	0
Clostridium_sensu_stricto_13	0	0	0	0	0	0	11	1	0	0	0	0	0	0	0	0	0	0
Clostridium_sensu_stricto_16	0	0	0	0	0	0	8	1	0	0	0	0	0	0	0	0	0	0
Clostridium_sensu_stricto_3	0	0	0	0	0	0	0	0	0	0	1	1	0	0	0	0	0	0
Clostridium_sensu_stricto_6	0	0	0	0	0	0	0	0	1	1	3	1	0	0	0	0	0	0
Clostridium_sensu_stricto_7	0	0	0	0	0	0	52	1	0	0	0	0	3	1	0	0	0	0
Collinsella	0	0	0	0	0	0	9	2	0	0	0	0	1	1	0	0	0	0
Coprococcus	0	0	137	6	7	4	20	2	7	3	5	3	2	1	10	3	12	3
Corynebacterium	0	0	0	0	1	1	8	2	1	1	1	1	3	2	1	1	0	0
CPla-4_termite_group	1	1	0	0	21	6	129	1	8287	28	7718	13	2	1	24	3	19	1
Dehalobacterium	0	0	3	3	28	9	0	0	15	9	16	6	8	1	12	10	23	15
Desulfomicrobium	1	1	0	0	0	0	0	0	0	0	0	0	0	0	1	1	0	0
Desulfovibrio	0	0	88	2	18	4	0	0	56	4	135	3	3	1	211	3	223	3
dgA-11_gut_group	0	0	92	4	238	6	0	0	20	4	52	3	0	0	151	4	137	4
Dietzia	0	0	0	0	0	0	7	1	0	0	0	0	0	0	0	0	0	0

Dorea	0	0	41	5	28	2	0	0	45	3	30	3	0	0	53	2	41	4
Edwardsiella	0	0	0	0	2	1	0	0	0	0	0	0	0	0	0	0	0	0
Elusimicrobium	0	0	38	3	0	0	0	0	35	2	84	4	0	0	36	3	38	3
Enhydrobacter	0	0	0	0	2	1	28	1	0	0	0	0	2	1	0	0	0	0
Enterococcus	84	1	0	0	1	1	0	0	0	0	0	0	0	0	1	1	0	0
Enterorhabdus	1	1	1	1	1	1	11	1	15	2	10	1	7	4	2	1	0	0
Epulopiscium	0	0	0	0	2	1	0	0	0	0	0	0	5	1	0	0	0	0
Escherichia-Shigella	26295	4	1	1	167	1	455	1	2	1	0	0	28	1	0	0	0	0
Eubacterium	0	0	1	1	3	1	0	0	0	0	2	1	4	1	2	1	1	1
Faecalibacterium	0	0	8	1	1	1	0	0	1	1	8	1	0	0	2	1	3	1
Fibrobacter	0	0	0	0	6	1	3	2	208	5	394	3	8	1	7	1	18	2
Flavobacterium	1	1	0	0	0	0	26	2	0	0	0	0	1	1	0	0	0	0
Flavonifractor	0	0	4	1	22	1	0	0	2	1	9	1	0	0	16	1	62	1
Fluviicola	0	0	1	1	0	0	3	1	0	0	0	0	0	0	0	0	0	0
Fusobacterium	0	0	0	0	2	2	0	0	0	0	0	0	0	0	0	0	0	0
Gelria	0	0	0	0	0	0	0	0	0	0	1	1	0	0	5	1	13	1
Gordonibacter	0	0	0	0	0	0	5	1	0	0	0	0	2	1	0	0	0	0
Gracilibacillus	0	0	0	0	0	0	45	1	0	0	0	0	0	0	0	0	0	0
Granulicatella	0	0	0	0	0	0	64	1	0	0	0	0	8	1	0	0	0	0
Halocella	0	0	0	0	0	0	18	1	0	0	0	0	0	0	0	0	0	0
Helicobacter	0	0	1	1	3	1	0	0	0	0	0	0	0	0	0	0	0	0
Hespellia	0	0	0	0	0	0	0	0	1	1	0	0	0	0	0	0	0	0
hgcI_clade	0	0	0	0	0	0	2	2	0	0	0	0	0	0	0	0	0	0
Holdemania	2	2	2	2	58	6	87	3	6	4	5	3	96	5	20	1	15	2
Howardella	1	1	0	0	0	0	0	0	0	0	1	1	0	0	0	0	0	0
Hydrogenoanaerobacterium	0	0	4	1	13	1	0	0	0	0	2	1	0	0	8	4	7	2
Hydrotalea	1	1	0	0	0	0	82	1	0	0	0	0	17	1	0	0	0	0
Iamia	0	0	0	0	0	0	3	1	0	0	0	0	0	0	0	0	0	0
Incertae_Sedis	106	15	2408	143	4348	238	3030	36	1634	161	1519	128	6486	43	2208	186	2271	171
Intestinimonas	0	0	35	3	18	3	0	0	40	4	21	4	1	1	125	6	77	6
Kroppenstedtia	0	0	0	0	0	0	7	2	0	0	0	0	0	0	0	0	0	0
Lachnospira	0	0	1	1	1	1	0	0	1	1	0	0	3	1	0	0	0	0
Lactobacillus	11461	10	2	2	0	0	205	3	1	1	1	1	0	0	1	1	0	0
Limnohabitans	1	1	0	0	0	0	0	0	0	0	0	0	0	0	0	0	0	0
Lysinibacillus	0	0	1	1	43	2	10	1	0	0	0	0	3	1	0	0	1	1

M2PT2-76_termite_group	0	0	0	0	10	1	0	0	4	1	3	1	0	0	25	1	13	1
Malikia	2	1	0	0	0	0	0	0	0	0	0	0	0	0	0	0	0	0
Marinomonas	0	0	0	0	0	0	24	1	0	0	0	0	2	1	0	0	0	0
Marvinbryantia	1	1	17	2	1	1	0	0	5	2	4	1	39	4	2	2	5	2
Megamonas	2	1	0	0	0	0	0	0	0	0	0	0	0	0	0	0	0	0
Megasphaera	7	1	0	0	0	0	0	0	0	0	0	0	0	0	0	0	0	0
Methylosinus	1	1	0	0	0	0	0	0	0	0	0	0	0	0	0	0	0	0
Micrococcus	1	1	0	0	0	0	14	1	0	0	0	0	4	1	0	0	0	0
Mogibacterium	22	3	0	0	39	5	411	6	6	2	7	3	973	9	8	3	3	3
Moryella	0	0	1	1	2	1	0	0	1	1	1	1	0	0	0	0	0	0
Mucispirillum	0	0	0	0	4	2	0	0	6	2	23	2	0	0	2	1	4	1
Mycoplasma	14	1	0	0	4	1	608	2	2	1	0	0	136	3	1	1	2	1
Neisseria	1	1	0	0	0	0	0	0	0	0	0	0	2	1	0	0	0	0
Nitrospira	0	0	1	1	0	0	0	0	0	0	0	0	0	0	0	0	0	0
Novosphingobium	0	0	0	0	0	0	11	2	0	0	0	0	0	0	0	0	0	0
Odoribacter	0	0	0	0	142	2	0	0	3	1	4	1	0	0	0	0	0	0
Olsenella	0	0	0	0	0	0	131	2	4	2	4	3	0	0	0	0	1	1
Oribacterium	0	0	1	1	0	0	42	2	5	3	8	6	1	1	0	0	0	0
Oscillibacter	0	0	407	13	157	12	13	1	88	13	159	12	0	0	336	14	281	17
Oscillospira	0	0	3	3	1	1	0	0	0	0	1	1	0	0	1	1	1	1
p-1088-a5_gut_group	2	2	0	0	54	4	0	0	178	4	315	7	42	1	71	3	52	1
Paludibacter	0	0	0	0	10	1	0	0	0	0	0	0	0	0	0	0	0	0
Papillibacter	0	0	0	0	6	4	51	2	165	5	137	5	2	1	2	2	0	0
Parabacteroides	0	0	23	1	222	2	3	1	36	2	90	2	0	0	1	1	2	1
Paracoccus	0	0	0	0	0	0	3	1	0	0	0	0	4	1	0	0	0	0
Paraprevotella	0	0	252	1	226	3	0	0	540	1	968	1	0	0	0	0	0	0
Parasporobacterium	0	0	0	0	0	0	0	0	1	1	0	0	0	0	0	0	0	0
Parasutterella	0	0	0	0	1	1	0	0	0	0	0	0	0	0	0	0	0	0
Parvibacter	0	0	1	1	0	0	0	0	3	1	0	0	0	0	0	0	0	0
Pelomonas	1	1	0	0	1	1	405	1	0	0	0	0	13	1	0	0	0	0
Pelospora	0	0	0	0	0	0	0	0	1	1	2	1	0	0	1	1	0	0
Peptostreptococcus	3	1	0	0	0	0	0	0	0	0	0	0	1	1	0	0	0	0
Phascolarctobacterium	0	0	311	8	18	1	0	0	84	3	180	4	0	0	235	4	237	3
Phocaeicola	0	0	144	10	45	4	0	0	54	3	62	4	1	1	3665	17	3406	13
Photobacterium	0	0	0	0	0	0	39	1	0	0	0	0	5	1	0	0	0	0

Pirellula	0	0	0	0	0	0	0	0	1	1	4	2	23	4	1	1	0	0
Plesiomonas	0	0	0	0	7	1	0	0	0	0	0	0	0	0	0	0	0	0
Polynucleobacter	0	0	1	1	1	1	0	0	0	0	0	0	0	0	0	0	0	0
possible_genus_Sk018	0	0	0	0	0	0	0	0	0	0	1	1	0	0	1	1	0	0
Prevotella	2	2	15	3	35	7	147	8	1143	24	1526	19	18	2	10	3	13	1
Propionibacterium	10	1	0	0	3	1	118	2	0	0	1	1	16	2	2	1	0	0
Proteus	1	1	0	0	0	0	1	1	0	0	0	0	0	0	0	0	0	0
Pseudobutyrivibrio	1	1	79	2	34	2	140	1	1	1	4	2	181	1	113	2	89	2
Pseudoflavonifractor	0	0	2	2	1	1	0	0	0	0	0	0	0	0	1	1	1	1
Pseudomonas	0	0	0	0	0	0	43	2	0	0	0	0	17	2	0	0	0	0
Pseudoramibacter	0	0	0	0	0	0	14	1	1	1	1	1	0	0	0	0	0	0
Psychrobacter	1	1	0	0	60	1	76	1	0	0	0	0	2	1	0	0	0	0
Pyramidobacter	0	0	0	0	0	0	25	2	0	0	0	0	2	1	0	0	0	0
Ralstonia	0	0	0	0	0	0	214	1	0	0	0	0	2	1	0	0	0	0
RC9_gut_group	2	2	3564	63	758	39	23	2	578	29	861	34	0	0	1273	28	1063	25
Reyranella	0	0	0	0	0	0	13	1	0	0	0	0	0	0	0	0	0	0
Rhizobium	0	0	0	0	0	0	0	0	0	0	0	0	1	1	0	0	0	0
Rhodococcus	0	0	0	0	1	1	23	1	0	0	0	0	0	0	0	0	0	0
Rikenella	0	0	0	0	0	0	0	0	4	2	2	2	0	0	0	0	0	0
Roseburia	0	0	10	2	7	2	25	2	2	2	24	2	5	1	2	2	6	1
Rothia	2	2	0	0	2	1	0	0	0	0	0	0	1	1	0	0	0	0
Rubrobacter	0	0	0	0	0	0	10	1	0	0	0	0	3	1	0	0	0	0
Ruminococcus	117	5	863	24	195	21	497	8	164	16	353	15	125	10	78	19	118	18
Saccharofermentans	27	3	12	1	21	2	4485	9	543	10	435	9	2050	10	36	1	18	1
Schwartzia	0	0	0	0	0	0	0	0	1	1	7	1	0	0	0	0	0	0
Selenomonas	0	0	0	0	0	0	0	0	1	1	1	1	1	1	0	0	0	0
Sharpea	0	0	0	0	0	0	6	1	0	0	0	0	0	0	0	0	0	0
Shuttleworthia	0	0	0	0	0	0	0	0	2	2	2	1	0	0	0	0	0	0
Slackia	0	0	0	0	1	1	6	1	0	0	0	0	5	1	0	0	0	0
Solibacillus	0	0	0	0	1	1	33	1	0	0	0	0	0	0	0	0	0	0
Solobacterium	52	8	7	3	37	10	837	13	4	4	4	3	1970	20	40	8	47	7
Spirochaeta	0	0	9	2	12	3	0	0	26	3	61	2	0	0	0	0	0	0
Sporobacter	0	0	580	11	131	7	48	2	158	14	121	8	0	0	78	6	74	9
Staphylococcus	2	1	0	0	0	0	83	1	0	0	0	0	3	2	0	0	0	0
Stenotrophomonas	1	1	0	0	0	0	24	1	0	0	0	0	0	0	0	0	0	0

Streptococcus	0	0	0	0	0	0	17	2	0	0	0	0	18	3	1	1	0	0
Succiniclasticum	0	0	0	0	0	0	5	1	41	1	70	2	0	0	0	0	1	1
Succinivibrio	0	0	169	3	0	0	0	0	90	3	812	7	0	0	0	0	0	0
Sulfurimonas	0	0	0	0	0	0	0	0	0	0	0	0	1	1	0	0	0	0
Sutterella	0	0	0	0	0	0	0	0	1	1	1	1	0	0	0	0	1	1
Syntrophococcus	1	1	1	1	5	2	23	4	7	4	3	1	174	7	3	3	3	2
Terrimonas	0	0	0	0	0	0	7	1	0	0	0	0	0	0	0	0	0	0
Thalassospira	0	0	1	1	0	0	0	0	0	0	0	0	57	2	26	2	16	1
Thermus	1	1	0	0	0	0	11	1	0	0	0	0	0	0	0	0	0	0
Tissierella	0	0	0	0	0	0	10	1	0	0	0	0	0	0	0	0	0	0
Treponema	0	0	1402	18	211	13	17	2	119	18	234	21	0	0	85	3	30	3
Turicibacter	0	0	25	3	16	1	476	3	4	1	9	2	59	1	78	2	29	2
unclassified	465	138	17302	1012	20907	1425	11602	288	15208	1197	16474	900	9860	370	15723	1467	15853	1358
Undibacterium	0	0	0	0	0	0	5	1	0	0	0	0	0	0	0	0	0	0
Ureaplasma	0	0	0	0	0	0	378	3	0	0	1	1	0	0	0	0	0	0
Veillonella	0	0	0	0	0	0	41	1	0	0	0	0	0	0	0	0	0	0
Victivallis	0	0	8	5	19	8	382	5	197	23	372	25	49	1	527	22	496	18
Yonghaparkia	2	1	0	0	0	0	37	1	0	0	0	0	0	0	0	0	0	0
