# Supplementary material for: A Metagenomics Investigation of Carbohydrate-Active Enzymes along the Gastrointestinal Tract of Saudi Sheep
Source: Front Microbiol. 2017 Apr 20;8:666. doi: 10.3389/fmicb.2017.00666 (PMC5397404; doi:10.3389/fmicb.2017.00666)
Supplement: Supplementary Table 4 — Abundance (number of 16S rRNA sequences) and diversity (number of unique OTUs) across the three sheep and the various intestinal subsites at the phylum level. [file Table4.PDF]

Supplementary Table 4. Abundance (number of 16S rRNA sequences) and diversity (number of unique OTUs) across the three sheep and the various intestinal subsites at the phylum level

	Najdi sheep						Noiami sheep						Harrei sheep					
	Small intestine		Large intestine		Rectum		Small intestine		Large intestine		Rectum		Small intestine		Large intestine		Rectum	
**Order**	Seq	OTUs	Seq	OTUs	Seq	OTUs	Seq	OTUs	Seq	OTUs	Seq	OTUs	Seq	OTUs	Seq	OTUs	Seq	OTUs
Acidimicrobiales	0	0	0	0	0	0	3	1	0	0	0	0	0	0	0	0	0	0
Actinomycetales	1	1	0	0	0	0	0	0	0	0	0	0	0	0	0	0	0	0
Aeromonadales	0	0	171	5	0	0	23	2	91	4	812	7	0	0	0	0	0	0
Anaerolineales	0	0	0	0	2	2	0	0	0	0	0	0	2	2	0	0	0	0
Anaeroplasmatales	0	0	0	0	34	4	0	0	0	0	0	0	0	0	34	1	27	2
Bacillales	3	2	2	2	112	13	335	15	1	1	0	0	15	6	2	1	5	3
Bacteroidales	8	7	11209	191	5828	196	482	34	7852	175	12319	180	33	10	11161	154	12627	134
BG.g7	0	0	0	0	3	2	0	0	4	2	5	3	0	0	2	1	5	2
Bifidobacteriales	176	3	11	3	8	1	5056	10	16	3	4	2	537	4	8	1	0	0
Burkholderiales	4	3	2	2	4	4	635	6	1	1	1	1	15	2	0	0	1	1
Campylobacterales	0	0	8	3	6	3	0	0	13	1	71	1	1	1	615	3	979	7
Caulobacterales	0	0	0	0	1	1	1	1	0	0	0	0	2	2	0	0	0	0
Chlamydiales	0	0	0	0	45	3	0	0	0	0	0	0	0	0	4	1	0	0
Clostridiales	973	94	17170	845	19315	1152	14488	202	11355	976	10159	743	14153	262	13351	1067	13490	996
Coriobacteriales	11	7	9	8	99	18	453	26	106	31	68	19	202	27	20	8	18	11
Corynebacteriales	0	0	0	0	2	2	38	4	2	2	1	1	3	2	1	1	0	0
Deferribacterales	0	0	0	0	4	2	0	0	6	2	23	2	0	0	2	1	4	1
Desulfovibrionales	1	1	88	2	18	4	0	0	63	6	145	4	4	2	212	4	225	5
Desulfuromonadales	0	0	74	5	0	0	0	0	45	4	85	5	0	0	2	2	10	1
Elusimicrobiales	0	0	38	3	0	0	0	0	35	2	84	4	0	0	36	3	38	3
EMP-G18	0	0	6	2	0	0	0	0	0	0	0	0	0	0	8	2	6	3
Enterobacteriales	26317	21	1	1	177	4	456	2	2	1	0	0	31	2	1	1	0	0

Erysipelotrichales	75	15	80	18	223	36	1886	30	105	22	90	21	2355	40	190	28	128	23
Fibrobacterales	0	0	0	0	6	1	3	2	208	5	394	3	8	1	7	1	18	2
Flavobacteriales	3	2	1	1	1	1	29	3	1	1	0	0	3	2	0	0	0	0
Frankiales	0	0	0	0	0	0	2	2	0	0	0	0	0	0	0	0	0	0
Fusobacteriales	0	0	0	0	109	5	0	0	0	0	0	0	0	0	0	0	0	0
Gastranaerophilales	42	19	10	4	4	4	398	31	11	7	26	12	2620	63	343	53	339	52
GR-WP33-30	0	0	0	0	0	0	0	0	1	1	0	0	0	0	0	0	0	0
Halanaerobiales	0	0	0	0	0	0	18	1	0	0	0	0	0	0	0	0	0	0
Lactobacillales	11549	14	3	3	1	1	286	6	1	1	1	1	26	4	3	3	0	0
Lineage_I_(Endomicrobia)	0	0	0	0	0	0	0	0	4	1	8	1	0	0	0	0	0	0
Methylococcales	0	0	0	0	0	0	0	0	0	0	1	1	0	0	0	0	0	0
Micrococcales	6	5	0	0	3	2	51	2	0	0	0	0	13	3	0	0	0	0
Mycoplasmatales	14	1	0	0	4	1	986	5	2	1	1	1	136	3	1	1	2	1
Myxococcales	0	0	0	0	0	0	0	0	0	0	0	0	0	0	0	0	3	1
NB1-n	0	0	6	3	154	13	0	0	18	5	9	3	0	0	323	30	241	24
Neisseriales	2	2	0	0	0	0	0	0	0	0	0	0	2	1	0	0	0	0
Nitrospirales	0	0	1	1	0	0	0	0	0	0	0	0	0	0	0	0	0	0
Obscuribacterales	0	0	0	0	1	1	2	2	0	0	0	0	0	0	0	0	0	0
Oceanospirillales	0	0	0	0	0	0	24	1	0	0	0	0	2	1	0	0	0	0
Oligosphaerales	0	0	30	1	2	1	0	0	109	4	246	8	21	2	8	2	1	1
Pasteurellales	0	0	0	0	2	1	0	0	0	0	0	0	0	0	0	0	0	0
Planctomycetales	4	4	0	0	75	10	129	1	8466	33	8037	22	67	6	96	7	71	2
Propionibacteriales	10	1	0	0	3	1	118	2	0	0	1	1	16	2	2	1	0	0
Pseudomonadales	1	1	0	0	62	2	155	5	0	0	0	0	21	4	0	0	0	0
RF9	8	4	40	17	71	32	3433	21	29	16	23	15	589	37	207	73	227	71
Rhizobiales	2	2	0	0	0	0	130	4	0	0	0	0	4	3	0	0	1	1
Rhodobacterales	0	0	0	0	0	0	3	1	0	0	0	0	5	2	0	0	0	0

Rhodospirillales	0	0	1	1	0	0	13	1	0	0	2	1	57	2	26	2	16	1
Rickettsiales	0	0	1	1	3	2	7	2	0	0	0	0	0	0	0	0	0	0
RS-B22	0	0	0	0	0	0	0	0	0	0	0	0	0	0	1	1	1	1
Rubrobacterales	0	0	0	0	0	0	10	1	0	0	0	0	3	1	0	0	0	0
SAR11_clade	0	0	0	0	0	0	0	0	0	0	0	0	1	1	0	0	0	0
Selenomonadales	12	4	319	12	18	1	54	3	128	7	260	10	3	2	240	5	243	7
Sphingobacteriales	1	1	0	0	0	0	90	3	0	0	0	0	17	1	0	0	0	0
Sphingomonadales	0	0	0	0	0	0	35	3	0	0	0	0	0	0	0	0	0	0
Spirochaetales	0	0	1411	20	241	18	17	2	149	22	300	25	0	0	110	4	43	4
Subgroup_3	0	0	0	0	0	0	1	1	0	0	0	0	0	0	0	0	0	0
Subgroup_6	3	1	0	0	0	0	0	0	0	0	0	0	0	0	0	0	0	0
Synergistales	0	0	0	0	1	1	25	2	0	0	2	1	2	1	0	0	0	0
Thermales	1	1	0	0	0	0	11	1	0	0	0	0	0	0	0	0	0	0
Thermoanaerobacterales	0	0	0	0	0	0	0	0	0	0	1	1	0	0	5	1	13	1
unclassified	58	35	2278	322	3450	471	2292	82	2349	375	2327	271	3173	106	4277	494	4166	461
Unknown_Order	363	21	191	14	359	22	5364	23	41	8	54	7	13247	74	766	21	1063	22
vadinHA64	0	0	1	1	1	1	0	0	17	1	22	1	0	0	0	0	0	0
Verrucomicrobiales	0	0	71	3	2622	20	39	1	1704	22	992	9	1	1	190	5	112	5
Vibrionales	0	0	0	0	0	0	47	2	0	0	0	0	5	1	0	0	0	0
Victivallales	0	0	8	5	19	8	382	5	197	23	372	25	49	1	527	22	496	18
Xanthomonadales	1	1	0	0	0	0	24	1	0	0	0	0	0	0	0	0	0	0
