# Supplementary material for: A Metagenomics Investigation of Carbohydrate-Active Enzymes along the Gastrointestinal Tract of Saudi Sheep
Source: Front Microbiol. 2017 Apr 20;8:666. doi: 10.3389/fmicb.2017.00666 (PMC5397404; doi:10.3389/fmicb.2017.00666)
Supplement: Supplementary Table 5 — Number of CAZymes detected by family, for each animal and each subsite. [file Table5.PDF]

	sheep Najdei			sheep Noaymi			sheep Harrei		
	small intestine	large intestine	rectum	small intestine	large intestine	rectum	small intestine	large intestine	rectum
CBM2		1	2	5		3	5	1	
CBM3		2	1	3	5	4	9		
CBM4		14	1	2	8	5	7	1	
CBM5	8								
CBM6		14	7		47	54		14	13
CBM9		4	16		5	10		6	5
CBM11					1	1			
CBM12	1	1	6	3				4	7
CBM13		16	11	42	14	14	83	20	27
CBM14			9						
CBM16		1	7		1	1		2	3
CBM20		36	1		20	18		31	23
CBM22		1	4	4	6	5		2	2
CBM26	6	5						3	
CBM27		4							
CBM30					2	3			
CBM32		502	24	4	60	60		38	31
CBM34	6	18	5	4	3	4		1	
CBM35		15	1		17	12		10	13
CBM37				20	1			1	
CBM38		12	1		7	10		3	4
CBM40							3		
CBM41		2	1						1
CBM47					4	3			
CBM48	5	93	38	11	55	44	41	73	69
CBM50	88	57	16	13	20	37	35	75	52
CBM51		1	1	1	22	20	4	1	1
CBM54								1	
CBM57		3	1			2		3	2
CBM58								1	1
CBM6		14	7		47	54		14	13
CBM61		3	1	2	2	4	1	3	1
CBM62		22	1		2	5		4	4
CBM66						1			
CBM67		33	7	1	15	10	1	19	13
CBM72		6	1			1			
CE1	8	210	42	18	167	177	52	92	91
CE2	3	29	14	3	40	29	2	22	17
CE3			2		2	2			
CE4	14	145	119	27	126	94	61	184	148
CE5			1				18		
CE6		16	12	1	29	31		11	10
CE7		47	20	1	44	41	2	40	40
CE8	11	31	17	1	20	12	4	17	13
CE9	8	117	132	27	79	73	38	112	119
CE11	2	54	26	5	55	56	17	57	44
CE12		18	10	1	26	21	1	23	18

CE14	3	29	19	1	21	12	1	17	28
CE15		41	37	3	47	29	3	27	37
GH1	26	40	62	38	13	9	130	30	23
GH2	17	556	387	58	460	445	102	492	454
GH3	15	350	237	41	310	304	111	321	312
GH4	11	70	76	11	55	34	15	85	74
GH5	5	148	71	29	223	181	28	104	103
GH8	12	8	4	6	27	20	1	8	2
GH9		70	30	7	70	80	22	33	26
GH10		80	28	18	112	111	12	61	48
GH11			2			4	1	1	
GH13	68	805	515	101	548	447	261	627	578
GH15								2	2
GH16		70	37	6	94	82	9	55	50
GH17			1						
GH18	7	76	19	2	23	22	1	33	45
GH19		4		4	9	5			1
GH20	4	285	194	8	218	243	5	274	305
GH23	60	113	54	35	81	103	59	99	88
GH24	61	42	22	44	20	16	45	41	29
GH25	46	56	70	115	51	59	196	67	52
GH26		64	24	8	52	59	1	23	28
GH27		54	46	6	43	32	10	31	35
GH28		123	68	2	83	52	28	84	81
GH29		139	144	8	178	147	12	129	149
GH30		55	16	4	38	40	2	35	35
GH31	18	120	81	28	180	137	20	106	101
GH32	11	53	18	12	42	28	44	19	15
GH33		75	82	9	118	111	14	110	117
GH35		80	47	1	55	44	6	46	51
GH36	5	168	161	26	141	121	57	134	141
GH37	5	22	6	4	1	1		3	4
GH38	6	164	210	12	112	80	17	141	180
GH39		36	19	5	57	63	7	32	31
GH42	11	50	42	3	35	28	3	27	36
GH43	2	277	156	25	344	356	61	195	216
GH44		1	5		3	3			1
GH45					5	3			
GH47						1			
GH48		1				1	1		
GH50		5	4	1	25	19	4	36	13
GH51	6	63	65	8	134	105	15	52	75
GH52					1				
GH53		65	33	2	33	26		28	22
GH54					3	2			
GH55							1	6	2
GH57	1	40	21	9	51	39	100	56	37
GH59				1	1				
GH63	4	40	8	5	17	16	4	34	30
GH65	13	19	19	3	12	17		21	11

GH66		3			2	3		4	3
GH67		17	4	5	21	23	2	7	10
GH73	21	53	30	14	52	51	43	61	46
GH74		7	5	6	16	12		5	3
GH76		3	1		2			7	9
GH77	2	185	199	20	138	127	59	214	187
GH78		273	175	11	165	124	37	216	216
GH79		13	2		7	6		10	5
GH81		6			1	1			
GH84		31	42		42	26		35	41
GH85			2		2			7	3
GH87		2	1						1
GH88		39	34		19	20		45	35
GH89		26	68	2	52	55		71	51
GH91		1							
GH92		226	106	1	166	173	6	204	210
GH93		6		2	5	2		1	3
GH94		51	76	17	79	55	60	59	59
GH95		94	88	2	102	110	3	129	107
GH97		172	84	20	195	190	119	180	137
GH98					4	5			
GH101								1	
GH102	4		1	9	5	2	8		3
GH103	4			2	7	3	5	4	2
GH104	14								
GH105		37	24		24	29	6	41	60
GH106		54	32	3	88	72	21	55	56
GH108	6	5		18	1		5	5	6
GH109		73	52	3	56	57		78	53
GH110		52	51		36	32	2	44	39
GH111				1			8		
GH112	1	19	16		5	7	1	30	13
GH113		1	2	1	4	4	1	2	1
GH115		40	9	3	62	64	4	23	34
GH116		3	22		11	9	1	22	14
GH117		2	3			1		2	1
GH120	8	8	6	8	43	29	20	1	2
GH121		1							
GH123		29	20	3	23	27	12	44	43
GH125		40	22		16	22		32	23
GH127	9	69	82	17	51	52	14	76	49
GH128		7	1		3	4		2	2
GH129		6	5		7	6		6	7
GH130		45	33	5	46	40	2	57	54
GH133		37	10	4	23	30	5	39	28
GT1		21	21	17	30	25	29	19	31
GT2	78	733	444	283	911	866	717	742	749
GT3		68	28		36	42		36	27
GT4	108	737	467	165	778	649	568	700	633
GT5	2	125	86	26	92	71	52	107	115

GT6					3	3	4		
GT8	38	31	39	21	42	18	130	48	42
GT9	19	17	11	13	50	21	15	34	31
GT10		1	4	1	3		5	1	1
GT11	2	5	8	1	15	9	34	7	9
GT14	2	1	5	1		4	5		2
GT17		1						1	
GT19	2	50	38	7	48	57	20	54	51
GT20	3		1						
GT21					6	6			
GT22					1			1	
GT23		1						3	
GT25	1				8	10			
GT26	5	43	19	4	30	18	16	28	32
GT27								1	3
GT28	10	165	138	38	112	116	140	153	129
GT30	3	38	25	9	42	40	18	23	28
GT32	4	49	20	2	30	37	11	22	31
GT35	5	229	242	58	205	191	130	249	196
GT39	1	7	32	6	21	11	9	5	9
GT41					3				
GT44	11								1
GT51	25	106	84	42	137	107	109	82	92
GT55			3			1	3	1	
GT56	4			3				2	
GT60		1					3	2	1
GT66			1		2	2	3	9	9
GT73	4								
GT76			1			1			
GT81			1			1	1		
GT83	2	1	6	6	17	15	4	12	5
GT84		6	16		16	15		13	9
PL1		38	12	1	30	30	1	40	33
PL2		1							
PL6		2	1		1				
PL8		17	5		1	1		8	6
PL9		1	3		7	8	5	7	2
PL10		9	6	2	1	5		7	8
PL11		8	11	2	29	35	1	11	13
PL12		11	5		20	7		15	15
PL13			1						
PL15		3	2					1	
PL17		1	1						
PL21		6	12		3	6		9	4
PL22		1	2		1		1	2	
PL23			16						
total	975	10360	6823	1793	9363	8505	4271	8874	8373
